# miR-125b-5p enhances chemotherapy sensitivity to cisplatin by down-regulating Bcl2 in gallbladder cancer

**DOI:** 10.1038/srep43109

**Published:** 2017-03-03

**Authors:** Dong Yang, Ming Zhan, Tao Chen, Wei Chen, Yunhe Zhang, Sunwang Xu, Jinchun Yan, Qihong Huang, Jian Wang

**Affiliations:** 1 Department of Biliary-Pancreatic Surgery, Renji Hospital, School of Medicine, Shanghai Jiao Tong University, 1630 Dongfang Road, Shanghai 200127, China; 2 Department of Radiation Oncology, Cancer Hospital of Fudan University, 270 Dong An Road, Shanghai 200032, China; 3 The Wistar Institute, 3601 Spruce Street, Philadelphia, PA 19104, USA

## Abstract

Gallbladder cancer represents the most common malignancy of the biliary tract and is highly lethal with less than 5% overall 5-year survival rate. Chemotherapy remains the major treatment for late-stage patients. However, insensitivity to these chemotherapeutic agents including cisplatin is common. MicroRNAs (miRNAs) have been shown as modulators of drug resistance in many cancer types. We used genome-wide gene expression analysis in clinical samples to identify miR-125b-5p down-regulated in gallbladder cancer. miR-125b-5p up-regulation promoted cell death in gallbladder cancer cells in the presence of cisplatin. In contrast, knockdown of miR-125b-5p reduced cell death in gallbladder cancer cells treated with cisplatin. Up-regulation of miR-125b-5p significantly decreased tumor growth in combination with cisplatin in a mouse model. We identified Bcl2 as a direct target of miR-125b-5p which mediates the function of miR-125b-5p in gallbladder cancer. In clinical samples, miR-125b-5p was down-regulated in gallbladder cancer whereas Bcl2 was up-regulated and their expression was inversely correlated. Moreover, low miR-125b-5p expression or high expression of Bcl2 is correlated with poor prognosis in gallbladder cancer. Taken together, our findings indicate that miR-125b-5p is a potent chemotherapy sensitizer and may function as a new biomarker for the prognosis of gallbladder cancer patients.

Gallbladder cancer (GBC) is the most common biliary tract cancer in clinic worldwide^1^. Chemotherapies such as cisplatin (CDDP) remain the major treatment for patients with gallbladder cancer or cholangiocarcinoma^2^. However, resistance to chemotherapies leads to dismal prognosis^3^. The overall survival is less than 12 months even with the combination of cisplatin and gemcitabine^4^. Therefore, it is crucial for an extensive and thorough understanding of the molecular mechanisms of chemotherapy resistance in GBC.

MicroRNAs (miRNAs) are 20–22 nucleotide long molecules that function as post-transcriptional regulators. They bind directly to the 3′ untranslated regions (3′ UTRs) of target mRNAs and mediate their degradation. MicroRNAs have been shown to play important roles in cancer development including gallbladder cancer^5^,^6^,^7^. However, their functions in chemotherapy resistance are not well studied.

The Bcl2 family proteins are key regulatory components of the intracellular apoptosis pathway which is critical for cancer development^8^. Bcl2 is uniformly expressed in chronic lymphocytic leukemia and promotes leukemia cell survival^9^. Additionally, it has been documented that Bcl2 promotes cell migration and invasion in colorectal cancer^10^ and hepatocarcinoma cells^11^. However, whether and how Bcl2 is involved in the development of gallbladder cancer has not been studied.

We recently found that miR-125b-5p is significantly down-regulated in gallbladder cancer from genome-wide microRNA expression profiling in GBC and neighboring normal tissues. miR-125b-5p directly suppresses Bcl2 expression and increases the sensitivity of cisplatin treatment in gallbladder cancer cells and mouse models. Because miR-125b-5p was identified in clinical samples prior to adjuvant therapy, it contributes to intrinsic resistance to chemotherapy, rather than acquired resistance. The expression of miR-125b-5p and Bcl2 is inversely correlated and predicts prognosis. These results suggest novel therapeutic targets in gallbladder cancer treatment.

## Results

### Identification of gallbladder cancer-related miRNAs by genome-wide miRNA expression analysis in clinical samples

**Figure 1:**
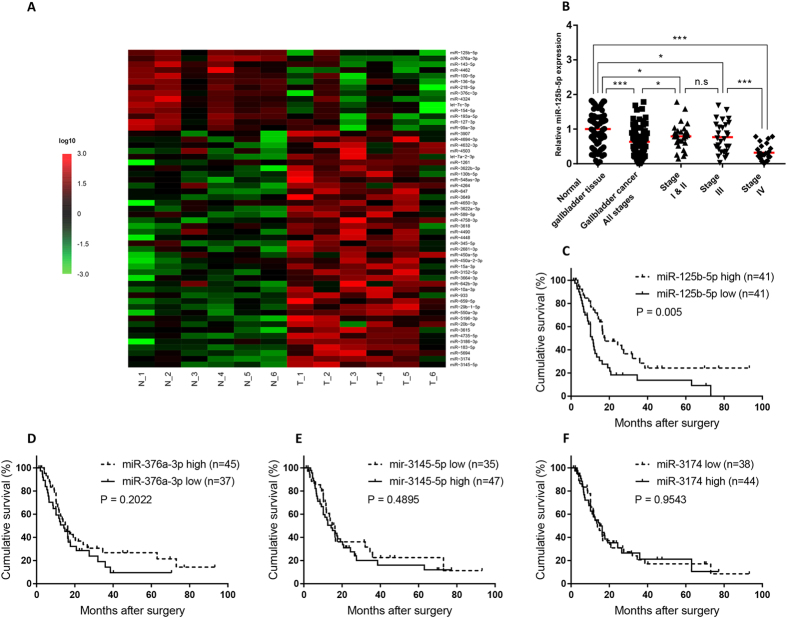
miR-125b-5p is down-regulated in human gallbladder cancer. (**A**) Heatmap demonstrating microRNA expression of six paired human gallbladder cancer and neighboring normal tissues. The heatmap analysis was performed by R software with gplots package (p value < 0.05 and log10 fold change >2 or <0.5). (**B**) Validation of miR-125b-5p expression of different stages (All stages; Stage I&II; III; IV) in human gallbladder cancer and neighboring normal tissues. miR-125b-5p expression is down-regulated in clinical gallbladder cancer samples (*p < 0.05, ***p < 0.001). (**C**–**F**) Gallbladder cancer patient survival was analyzed by Kaplan-Meier analysis. P value was calculated using log-rank test. MicroRNA expression which exceeds the average level (mean value) is defined as “high” while microRNA expression which is less than the average level (mean value) is defined as “low”. (**C**) Lower miR-125b-5p expression in tumor is correlated with poor prognosis. (**D**) miR-376a-3p expression is not correlated with survival in gallbladder cancer. (**E**) miR-3145-5p expression is not correlated with survival in gallbladder cancer. (**F**) miR-3174 expression is not correlated with survival in gallbladder cancer.

To identify potential miRNAs involved in gallbladder cancer progression, we performed a genome-wide miRNA expression analysis in six pairs of gallbladder cancer samples and their neighboring normal tissues. The expression of sixty miRNAs was found to be significantly altered between cancer and neighboring normal tissues (Fig. 1A). Among the top miRNA candidates, miR-125b-5p is highly down-regulated in tumor tissues than normal tissues. miR-125b-5p has been shown to be involved in endometriosis, myocardial infarction and chronic hepatitis B^12^,^13^,^14^, but its functions in cancer development have not been studied.

To validate the down-regulation of miR-125b-5p expression in gallbladder cancer, we performed qPCR in 82 paired human gallbladder cancer and their corresponding neighboring normal tissues. The expression of miR-125b-5p was down-regulated in cancer tissues when compared with normal tissues (Fig. 1B). To determine whether the expression of microRNA candidates is correlated with prognosis, we selected two most down-regulated microRNAs, miR-125b-5p and miR-376a-3p (Fig. 1A), and two most up-regulated microRNAs, miR-3145-5p and miR-3174 (Fig. 1A), in gallbladder cancer to determine the correlation of their expressions and survival in clinical gallbladder cancer samples. Low expression of miR-125b-5p is correlated with poor prognosis (Fig. 1C), whereas the expression of miR-376a-3p (Fig. 1D), miR-3145-5p (Fig. 1E), and miR-3174 (Fig. 1F) is not correlated with prognosis. These data suggested that miR-125b-5p expression is significantly altered in gallbladder cancer and its low expression is correlated with poor prognosis.

### miR-125b-5p sensitize gallbladder cancer to cisplatin treatment

**Figure 2:**
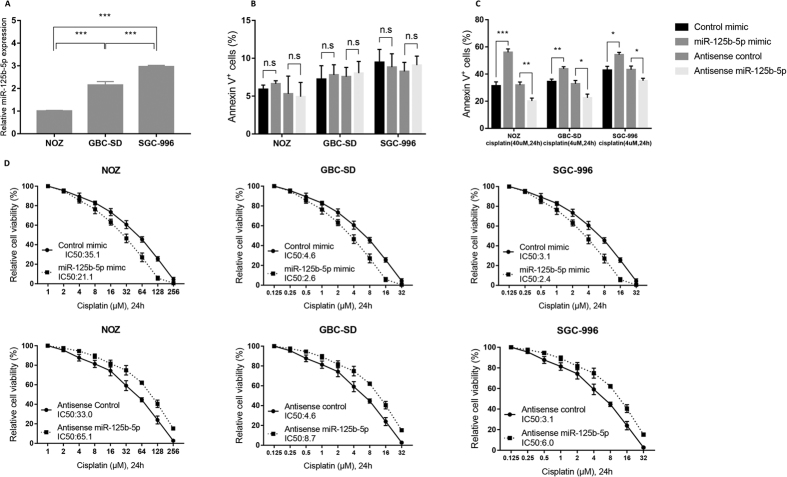
miR-125b-5p increases cytotoxic sensitivity to cisplatin treatment in human gallbladder cancer cells. (**A**) miR-125b-5p expression in human gallbladder cancer cell lines, NOZ, GBC-SD, SGC-996, was quantified by qPCR. (**B**–**C**) NOZ, GBC-SD, SGC-996 cells were transfected with miR-125b-5p mimic or control mimic oligos, antisense miR-125b-5p or control anti-sense oligos. Cells were treated with cisplatin for 24 hours or with no treatment. Apoptosis were measured using Annexin V/PI staining. Student t test was used to calculate p value. *P < 0.05; **P < 0.01; ***P < 0.001; n.s, no significance. (**B**) miR-125b-5p mimics or antisense oligos did not affect cell death. (**C**) miR-125b-5p mimics sensitized gallbladder cells to cisplatin treatment and miR-125b-5p antisense oligos increased resistance to cisplatin treatment in gallbladder cancer cells. (**D**) IC50s of NOZ, GBC-SD, SGC-996 cells treated with cisplatin were calculated using cell viability data in Fig. 2B. Ectopic expression of miR-125b-5p decreased IC50 when compared with that of cells transfected with control oligos. Inhibition of miR-125b-5p by anti-sense oligos increased IC50 when compared with that of cells transfected with control anti-sense oligos.

miR-125b-5p expression was determined in three gallbladder cancer cell lines NOZ, GBC-SD, and SGC-996 by qPCR. NOZ has the lowest miR-125b-5p expression whereas SGC-996 has the highest (Fig. 2A). It has been shown that NOZ is highly resistant to chemotherapy treatment^15^, thus we determined whether miR-125b-5p played any role in the sensitivity of cisplatin treatment in gallbladder cancer. miR-125b-5p mimics, antisense oligos, control mimics, and control antisense oligos were transfected into NOZ, GBC-SD, and SGC-996 cells which were subsequently treated with cisplatin or with no treatment. Cell death was measured by Annexin V staining and FACS analysis. Over-expression or knock-down of miR-125b-5p did not affect cell death (Fig. 2B). Overexpression of miR-125b-5p in these cells led to significant cell death when cells were treated with cisplatin (Fig. 2C). In contrast, downregulation of miR-125b-5p led to resistance in cells treated with cisplatin (Fig. 2C). IC50s of cisplatin were reduced in NOZ, GBC-SD, and SGC-996 cells transfected with miR-125b-5p mimics (Fig. 2D), but increased in these cells transfected with miR-125b-5p antisense oligos (Fig. 2D). These results indicated that miR-125b-5p is involved in cisplatin sensitivity in gallbladder cancer cells.

### Bcl2 is a direct target of miR-125b-5p and mediates its function in gallbladder cancer

**Figure 3:**
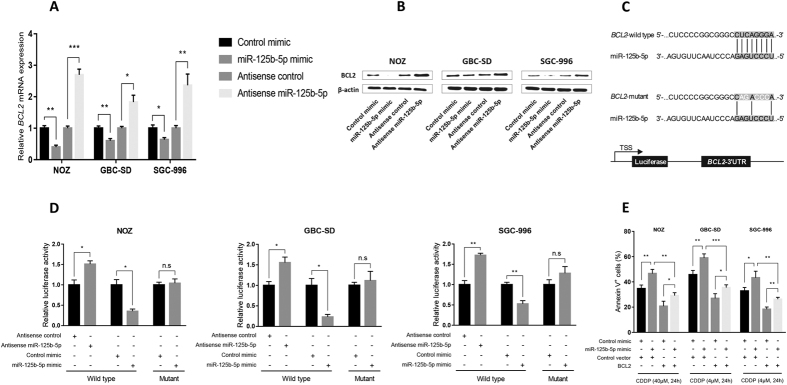
Bcl2 expression is directly suppressed by miR-125b-5p. (**A**,**B**) Human gallbladder cancer cell lines, NOZ, GBC-SD, SGC-996, were tranfected with miR-125b-5p mimics, or control mimics, anti-sense miR-125b-5p, or control anti-sense oligos. Bcl2 mRNA expression in these cells were quantified by qPCR (**A**) and Bcl2 protein expression were measured by immunoblotting (**B**). (**C**) Bcl2-luciferase reporter plasmid construction. Seed sequence of miR-125b-5p targeting Bcl2 was indicted in grey. Mutant sequence of the reporter plasmid was indicted in white. (**D**) NOZ, GBC-SD, SGC-996 cells were transfected with wildtype or mutant Bcl2-luciferase reporter plasmid and miR-125b-5p mimics or control mimics or miR-125b-5p anti-sense oligos or control anti-sense oligos. Firefly luciferase activity was measured and normalized to the Renilla luciferase activity. (**E**) Bcl2 mediates the function of miR-125b-5p in apoptosis. NOZ, GBC-SD, SGC-996 cells were transfected with a Bcl2 cDNA plasmid without the 3′UTR and miR-125b-5p mimics or control mimics. Apoptosis were measured using Annexin V/PI staining. Student t test was used to calculate p value. *P < 0.05; **P < 0.01; ***P < 0.001.

To identify the direct targets of miR-125b-5p, we used microRNA target prediction program TargetScan 7.0^16^ and find Bcl2 is one of the potential targets which contain miR-125b-5p binding site at its 3′UTR region. Bcl2 has been shown to be involved in cell death^17^,^18^ that is highly relevant to the cisplatin sensitivity phenotype. Immunoblotting was used to determine the expression of Bcl2 in gallbladder cancer cells. Introduction of miR-125b-5p mimics in NOZ, GBC-SD, and SGC-996 cells substantially decreased Bcl2 protein expression whereas miR-125b-5p antisense oligos increased Bcl2 expression (Fig. 3A,B, Supplementary Figure 1). To test whether Bcl2 is a direct target of miR-125b-5p, we constructed reporter plasmids containing the 3′UTR of Bcl2 (Fig. 3C). Co-transfection of miR-125b-5p and the reporter plasmids showed that the introduction of miR-125b-5p significantly suppressed the expression of a luciferase gene containing the 3′UTR of Bcl2 but not the one containing the 3′UTR of Bcl2 with the mutation in the miR-125b-5p binding site (Fig. 3D). Conversely, knockdown of miR-125b-5p by antisense oligos increased the luciferase signal containing 3′UTR of Bcl2 but not 3′UTR of Bcl2 with mutant miR-125b-5p binding site (Fig. 3D). These results suggested that Bcl2 is directly regulated by miR-125b-5p.

To determine whether Bcl2 mediates the function of miR-125b-5p in cisplatin sensitivity, we co-transfected miR-125b-5p and Bcl2 cDNA without the 3′UTR which contains the miR-125b-5p binding site into NOZ, GBC-SD, and SGC-996 cells. Introduction of both miR-125b-5p and Bcl2 significantly reduced cell death in these cells treated with cisplatin when compared with miR-125b-5p and a control vector (Fig. 3E). These results suggested that Bcl2 is a critical mediator in the cisplatin sensitivity function of miR-125b-5p.

### miR-125b-5p enhances cisplatin therapy in a mouse model

**Figure 4:**
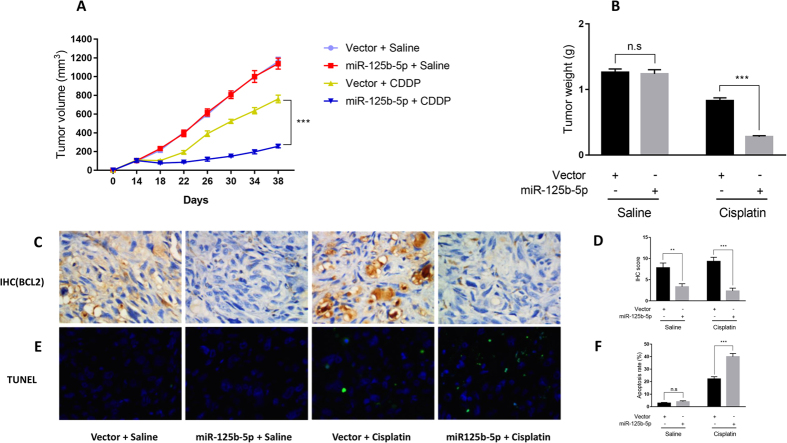
miR-125b-5p sensitize human gallbladder cancer cells to cisplatin treatment in a mouse model. (**A**,**B**) Human gallbladder cancer GBC-SD cells expressing miR-125b-5p or a control vector were transplanted in mice subcutaneously. Mice were treated with cisplatin or saline. Tumor volume (**A**) and tumor weight (**B**) were measured. P value was determined by student t test. **P < 0.01; ***P < 0.01; n.s, no significance. Tumor growth was significantly suppressed in mice expressing miR-125b-5p treated with cisplatin. (**C**,**D**) Representative images of Bcl2 expression in tumor tissues in mouse model (**C**). Bcl2 expression was determined using immunohistochemistry (**D**). Bcl2 expression was decreased in tumors expressing miR-125b-5p. (**E,F**) Representative images of apoptosis in tumor tissues in mouse model (**E**). Apoptosis was determined using TUNEL staining (**F**). Apoptosis was increased in tumor cells expressing miR-125b-5p and treated with cisplatin.

To further confirm the function of miR-125b-5p in cisplatin sensitivity, we transplanted GBC-SD cells into mice which were then treated with cisplatin or saline control. Tumor volume was measured to determine tumor growth. Introduction of miR-125b-5p did not affect tumor growth *in vivo* (Fig. 4A,B). Bcl2 expression was decreased in tumor cells with miR-125b-5p (Fig. 4C,D), which further confirmed that miR-125b-5p down-regulates Bcl2. Apoptosis of the tumor cells were similar in cells with or without miR-125b-5p overexpression (Fig. 4E,F), which is consistent with our observation that miR-125b-5p alone does not affect cell death or proliferation *in vitro*. miR-125b-5p markedly sensitize tumor cells to cisplatin treatment (Fig. 4A,B). Bcl2 expression in the tumor cells were further decreased in cells with miR-125b-5p and cisplatin treatment (Fig. 4C,D). Apoptosis of transplanted tumor cells were significantly increased in cells which were introduced with miR-125b-5p and treated with cisplatin when compared with cells with a control vector and cisplatin treatment (Fig. 4E,F). Taken together, these results further confirmed the function of miR-125b-5p in cisplatin sensitivity *in vivo*.

### miR-125b-5p and Bcl2 expressions are biomarkers for prognosis in human gallbladder cancer

**Figure 5:**
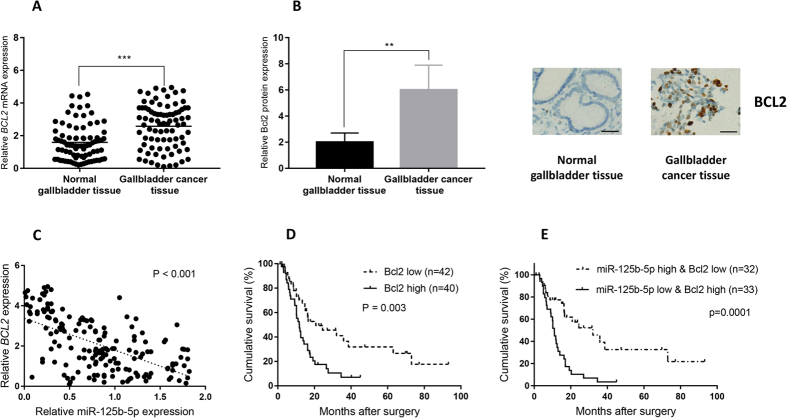
miR-125b-5p and Bcl2 expression are biomarkers for prognosis in human gallbladder cancer tissues. (**A**,**B**) Bcl2 mRNA (**A**) expression and protein expression (**B**) in clinical gallbladder cancer tissues and neighboring normal tissues was determined by qPCR and immunohistochemistry. Bcl2 expression was up-regulated in gallbladder cancer tissues than neighboring normal tissues. (**C**) Correlation of miR-125b-5p expression and Bcl2 expression in human gallbladder cancer tissues was analyzed using Pearson’s correlation analysis. r = −0.594, P < 0.001. The expression of miR-125b-5p and Bcl2 were inversely correlated. (**D**,**E**) Gallbladder cancer patient survival was analyzed by Kaplan-Meier analysis. P value was calculated using log-rank test. Bcl2 expression which exceeds the average level (mean value) is defined as “high” while Bcl2 expression which is less than the average level (mean value) is defined as “low”. (**D**) Higher Bcl2 expression in tumors is correlated with poor prognosis. (**E**) Patients with both lower miR-125b-5p expression and higher Bcl2 expression in tumors exhibit poor prognosis.

We have shown that miR-125b-5p was down-regulated in gallbladder cancer samples (Fig. 1A,B) and suppressed Bcl2 expression *in vitro* and *in vivo*. To investigate whether miR-125b-5p negatively regulates Bcl2 in clinical samples, we determined the Bcl2 expression in human gallbladder cancer samples. The expression of Bcl2 is higher in gallbladder cancer tissues than in neighboring normal tissues (Fig. 5A). Immunohistochemistry staining of Bcl2 showed higher Bcl2 expression in gallbladder cancer when compared with that in neighboring normal tissues (Fig. 5B). The expression of miR-125b-5p and Bcl2 are inversely correlated in gallbladder cancer samples (Fig. 5C). Low miR-125b-5p expression is inversely correlated with survival (Fig. 1C). Interestingly, high Bcl2 expression is correlated with poor survival in gallbladder cancer (Fig. 5D). Moreover, the combination of miR-125b-5p low expression and Bcl2 high expression is highly correlated with poor prognosis in clinical samples. These results further supported the regulation of Bcl2 by miR-125b-5p and demonstrated the significance of miR-125b-5p and Bcl2 as biomarkers in gallbladder cancer progression.

## Discussion

Cisplatin resistance is common among gallbladder cancer patients with cisplatin treatment as adjuvant therapy. Discovery of molecules and therapies that sensitize cisplatin in gallbladder cancer is urgently needed. We used a genome-wide microRNA expression profiling to identify miR-125b-5p markedly down-regulated in gallbladder cancer. Lower miR-125b-5p expression in GBC is correlated with poor prognosis. Moreover, miR-125b-5p sensitizes gallbladder cancer cells to cisplatin treatment. Bcl2 is a direct target of miR-125b-5p and mediates its functions. These findings suggest a novel therapeutic target for gallbladder cancer.

Although microRNAs have been implicated in gallbladder cancer development^5^,^19^, their functions in the sensitization of chemotherapies have not been well studied. We recently found that miR-145 is up-regulated in gallbladder cancer tissues and sensitize cisplatin treatment by suppressing MRP1^20^. In this work, we found that miR-125b-5p is down-regulated in human gallbladder cancer and has different mechanisms to enhance cisplatin sensitivity. It contributes to intrinsic resistance to chemotherapy after surgery rather than acquired resistance. These results indicate that cisplatin resistance in gallbladder cancer is due to multiple mechanisms. Therapeutics that targeting multiple molecules is required to improve therapeutic efficiency of cisplatin in GBC.

In summary, we showed for the first time that miR-125b-5p-Bcl2 pathway is potentially a therapeutic target for gallbladder cancer. MicroRNA mimics have been developed for therapeutics^21^,^22^,^23^,^24^, although delivery remains a major issue for their use in the clinic. Bcl2 small molecule inhibitors have also been developed and are currently in clinical trials for chronic lymphocytic leukemia patients^25^,^26^. Although Bcl2 inhibitors are easier to be delivered in patients, miR-125b-5p mimics potentially have advantages in therapy because miR-125b-5p target molecules in addition to Bcl2 may also contribute to resistance. In combination with cisplatin, these therapeutics would potentially overcome cisplatin resistance and improve clinical outcomes in gallbladder cancer.

## Materials and Methods

### Tissue samples

Formalin-fixed, paraffin-embedded (FFPE) cancer tissues were collected from 82 patients harboring histologically-confirmed GBC who underwent surgical resection of the gallbladder and postoperative adjuvant chemotherapy at the Department of Pathology (Renji Hospital) from January 2004 to December 2013 retrospectively. Fresh GBC tissues and the neighboring noncancerous gallbladder tissues were also obtained from 82 GBC patients. All fresh tumor samples were collected immediately after the surgical removal and snap-frozen in liquid nitrogen, then stored at −80 °C until total RNA was extracted. Postoperative survival was calculated from time of surgery to time of last follow-up or death. The collection and analysis of patient samples were approved by the Ethical Committee of Renji Hospital, Shanghai Jiao Tong University School of Medicine, and written informed consent was obtained from all patients. All methods and experiments were carried out in accordance with the approved guidelines and regulations.

### miRNA microarray

Total RNA samples were spiked using the MicroRNA Spike-In Kit (Agilent Technologies) to assess the labeling and hybridization efficiencies. After the spiked total RNA was treated with alkaline calf intestine phosphatase, a labeling reaction was initiated with 100 ng of total RNA per sample. T4 RNA ligase, which incorporates cyanine 3-cytidine biphosphate (miRNA Complete Labeling and Hyb Kit; Agilent Technologies), was used to label the dephosphorylated RNA. The cyanine 3-labeled miRNA samples were subsequently prepared for one-color hybridization (miRNA Complete Labeling and Hyb Kit). The labeled miRNA samples were hybridized to the Agilent Human miRNA Microarray 8 × 60 K Release 16.0 (Release 16.0, 8 × 60 K format; Agilent Technologies) for 20 h at 55 °C. After washing the microarray slides with buffers with increasing stringency (Gene Expression Wash Buffers; Agilent Technologies), the slides were dried with acetonitrile. Fluorescent signal intensities were detected on an Agilent Microarray Scanner (Agilent Technologies) with the Scan Control A.8.4.1 Software (Agilent Technologies) and were extracted from the images using the Feature Extraction 10.7.3.1 Software (Agilent Technologies). All of the steps described above were performed according to the manufacturer’s instructions. The raw miRNAs microarray data were normalized using the GeneSpring GX software version 12.0 (Agilent Technologies). The signal values were transformed to the log base 10, and then quantile and percentile shift was applied to obtain an equal distribution of probe signal intensities. The comparative analysis of the gallbladder cancer and normal control group samples was performed using the t-test (p-values) and SAM (http://www-stat.stanford.edu/~tibs/SAM/). Compared with the expression level of the reference RNA, the miRNAs were described as differentially expressed if the p-values were <0.05, and the fold change (FC) was greater than 2 or less than 0.5.

### Cell culture

The human embryonic kidney 293 cells (HEK293FT) and two human GBC cell lines, NOZ and GBC-SD were maintained in Dulbecco’s Modified Eagle Medium (DMEM), Another human GBC cell line SGC- 996 was maintained in RPMI-1640 medium, with all media containing 10% fetal bovine serum (FBS) and antibiotics (Gibco, Grand Island, NY, USA). Cells were maintained at 37 °C in a humidified atmosphere consisting of 5% CO2. NOZ was purchased from the Health Science Research Resources Bank (Osaka, Japan). GBC-SD and SGC-996 cells were provided by the Academy of Life Sciences, Tongji University (Shanghai, China). HEK293FT cells were purchased from Invitrogen (MD, USA) and were used for adenovirus amplification. Cisplatin was dissolved in dimethyl sulfoxide (DMSO). GBC cells were treated with cisplatin (40 μM in NOZ and 4 μM in GBC-SD or SGC-996 cells) or control DMSO.

### Cell transfection

Human miR-125b-5p expression construct was generated by insertion of the coding sequence (CDS) of miR-125b-5p into pCDHCMV- MCS-EF1-copGFP (System Biosciences, CA, USA). Recombinant lentiviruses were produced by transient transfection of HEK293FTcells, along with package vectors, using Lipofectamine 2000 (Invitrogen). After transfection for 48 h, the viruses were harvested and viral titers were determined. Then, GBC-SD cells were infected with lentiviruses in the presence of 4 μg/ml polybrene (Sigma), followed by puromycin selection (2 μg/ml). Bcl2 expression vector was constructed by cloning Bcl2 cDNAinto a pcDNA 3.1 vector (Invitrogen). NOZ, GBC-SD and SGC-996 cells were transfected with the Bcl2 expression plasmid using Lipofectamine 2000 (Invitrogen) transfection reagent according to the protocols. The pcDNA3.1 empty vector was used as negative control (vector). The miR-125b-5p mimic, miR-125b-5p inhibitor, and siRNA of Bcl2 were purchased from GenePharma (Shanghai, China). Cells were cultured to 60–70% confluence in six-well plates and then transfected using Lipofectamine 2000 (Invitrogen).

### Quantitative

Real-time PCR analysis Total RNA and miRNA were isolated from fresh tissues and cells using TRIzol reagent (Invitrogen) and miRNeasy Mini Kit (Qiagen, Hilden, Germany), and miRNAs were extracted from FFPE samples using miRNeasy FFPE Kit (Qiagen) according to the manufacturer’s instructions. After synthesizing cDNAs with Reverse Transcriptase M-MLV kit, the expression levels of miR-125b-5p and Bcl2 were analyzed using SYBR Premix Ex Taq (Takara, Shiga, Japan) and run with Applied Biosystems ViiA™ 7 Real-Time PCR System (Applied Biosystems, Foster City, CA). Data were analyzed by 2−ΔΔCT method^27^ and presented relative to the expression of GAPDH for Bcl2 and in relation to the expression of small nuclear U6 RNA for miR-125b-5p. The primer sequences are listed in Supplementary Table 1.

### Cytotoxicity, cell apoptosis, cell proliferation assays

Cell viability (NOZ, GBC-SD and SGC-996) was identified by 3-(4, 5-dimethylthiazol-2-yl)-5-(3-carboxymethoxyphenyl)-2-(4- sulfophenyl)-2H-tetrazolium assay (MTS; Promega, Madison, WI, USA). Briefly, cells were plated (5 × 10^3^ cells/well) on 96-well plates and incubated overnight to allow cell attachment. Then, NOZ cells were treated with cisplatin at a series of concentrations of 1, 2, 4, 8, 16, 32, 64, 128 and 256 μM while GBC-SD and SGC-996 cells were treated with cisplatin at concentrations of 0.125, 0.25, 0.5, 1, 2, 4, 8, 16, and 32 μM for 24 h, respectively. Subsequently, the MTS reagent (20 μl) was added to each well, followed by incubation at 37 °C in a humidified, 5% CO2 atmosphere for 2 h. Finally, the absorbance was read at 490 nm by using a Synergy 2 (BioTek, VT, USA) plate reader. The cell viability was indicated as a percentage relative to control. Cell proliferation was also analyzed using MTS assay (Promega) when NOZ, GBC-SD and SGC-996 cells were seeded into a 96-well plate (1 × 10^3^ cells/well) and cultured for 72 h. Apoptosis of gallbladder cancer cell lines were analyzed using Annexin V/PI Apoptosis Detection Kit (BD Biosciences, MA, USA) according to the manufacturer’s instructions. Cells were seeded in six-well plates and grown to approximately 60% confluence, followed by treating with cisplatin (40 μM in NOZ and 4 μM in GBC-SD or SGC-996) for 48 h. Cells were harvested and incubated with Annexin V/PI for 15 min in the dark, followed by fluorescence-activated cell sorting (FACS) analysis. All the assays were carried out four times.

### Reporter vector constructs and dual luciferase reporter assay

The fragment from Bcl2-3′-UTR containing the predicted miR-125b-5p binding site was amplified by PCR and then cloned into a pmirGLO Dual-Luciferase miRNA Target Expression Vector (Promega) to form the reporter vector Bcl2-3′-UTR wild type. The putative binding site of miR-125b-5p in the Bcl2- 3′-UTR was mutated by using a site-directed mutagenesis kit from Fast Mutagenesis System (TransGen Biotech, Beijing, China), and the mutant reporter vector was named as Bcl2-3′-UTR mutant. The miR-125b-5p mimic and vector were co-transfected into 3 human gallbladder cancer cells, and Renilla luciferase reporter plasmid (pRL-TK) was also co-transfected as the internal reference. After transfection for 48 h, cells were lysed in passive lysing buffer, and then firefly and Renilla luciferase activities were analyzed using the Dual-Luciferase Reporter Assay System (Promega). The results of firefly luciferase activity were normalized to the Renilla luciferase activity.

### Western blot analysis

For protein isolation from NOZ, GBC-SD and SGC-996 cells, RIPA buffer supplemented with proteinase inhibitor cocktail was used. The protein concentration was determined using the BCA assay. Equal amounts of cell lysates were loaded on a 10% sodium dodecyl sulfate-polyacrylamide gel for electrophoresis (SDS-PAGE) and transferred to PVDF membranes (Millipore, IL, USA). The membranes were blocked for 1 h at room temperature using Tris-bufferred saline with 0.05% Tween 20 (TBST) and 5% skimmed milk, and then the following primary antibodies were applied overnight at 4 °C: anti-Bcl2 (Santa Cruz, CA, USA) and anti-β-actin (Sigma). After washing three times with TBST, the membranes were incubated with secondary antibody at room temperature for 2 h and washed again with TBST. Images of target proteins were detected by chemiluminescence HRP substrate kit (Millipore).

### Immunohistochemistry and terminal deoxynucleotidyl transferase dUTP nick end labeling assays

All specimens from patients and subcutaneous xenografts fixed in 10% buffered formalin were embedded in paraffin blocks. Consecutive 4-μm thick sections were analyzed using a standard immunohistochemistry protocol and stained by antibodies of Bcl2 (1:100, Santa Cruz). Positive staining cells were visualized by DAB systems and counterstained with hematoxylin. The stained sections were photographed and converted to a digital image using light microscopy equipped with camera (Olympus, Tokyo, Japan). The scoring of immunohistochemistry (IHC) is based upon the staining intensity (I) and the proportion of stained quantity (q) of tumor cells to obtain a final score (Q) defined as the product of I × q and was performed by two independent pathologists. The scoring system for I was 0 = negative, 1 = low, 2 = moderate, 3 = intense immunostaining; and for q was 0 = negative, 1 = 1–9% positive, 2 = 10–39% positive, 3 = 40–69% positive, and 4 = 70–100% positive cells. Cell apoptosis of xenograft sections was also detected by using *In Situ* Cell Death Detection Kit, POD (Roche, Basel, Switzerland) according to the manufacturer’s instruction. The sections were visualized with DAB and counterstained with hematoxylin. The number of terminal deoxynucleotidyl transferase dUTP nick end labeling (TUNEL)-positive cells was randomly counted in five fields, and the apoptosis index for each field was calculated as the percent of TUNEL-positive cells relative to the total cells.

### *In vivo* studies

Animal maintenance and experimental procedures were strictly performed following the guidelines of the Animal Care and Use Committee of Shanghai Jiao Tong University and approved by IACUC committee of Shanghai Jiao Tong University. A total 1 × 10^6^ GBC-SD/pcDNA3.1 miR-125b-5p or GBC-SD/pcDNA3.1 empty vector cells in 60 μl medium were subcutaneously transplanted into 4-week-old male nude mice of each group (group 1, vector; group 2, miR-125b-5p; group 3, vector + cisplatin; group 4, miR-125b-5p + cisplatin; n = 6/group). When the average tumor size reached approximately 0.1 cm^3^, cisplatin was administered via intraperitoneal injection at a dose of 4 mg/kg at Day 14 and Day 24. Saline was used as a control. Tumor volumes were examined using external caliper once every 4 days and were calculated based on the equation: V = (length × width^2^)/2^28^. All mice were sacrificed at the 44th day, and the tumors were dissected out for hematoxylin and eosin (H&E) staining, IHC staining, and TUNEL staining. All methods and experiments were carried out in accordance with the approved guidelines and regulations.

### Statistics

Data are expressed as mean ± SEM. Two-group comparisons were performed with unpaired two-tailed Student’s t test. Survival probabilities were determined using Kaplan-Meier analyses and compared by the log-rank test. Each experiment consisted of at least four replicates per condition. SPSS 17.0 software was used for all statistical analysis. P < 0.05 was considered statistically significant.

## Additional Information

**How to cite this article:** Yang, D. *et al*. miR-125b-5p enhances chemotherapy sensitivity to cisplatin by down-regulating Bcl2 in gallbladder cancer. *Sci. Rep.* **7**, 43109; doi: 10.1038/srep43109 (2017).

**Publisher's note:** Springer Nature remains neutral with regard to jurisdictional claims in published maps and institutional affiliations.

## Supplementary Material

Supplementary Information
