# Supplementary material for: miR-125b-5p enhances chemotherapy sensitivity to cisplatin by down-regulating Bcl2 in gallbladder cancer
Source: Sci Rep. 2017 Mar 3;7:43109. doi: 10.1038/srep43109 (PMC5335654; doi:10.1038/srep43109)
Supplement: Supplementary Information [file srep43109-s1.pdf]

miR-125b-5p enhances chemotherapy sensitivity to cisplatin by down-regulating Bcl2 in gallbladder cancer

Dong Yang, Ming Zhan, Tao Chen, Wei Chen, Yunhe Zhang, Sunwang Xu, Jinchun Yan, Qihong Huang, and Jian Wang

Supplementary Figure 1. Uncropped immunoblot gel of Bcl2 expression.

Supplementary Table 1. PCR primer sequences.

Supplementary Figure 1

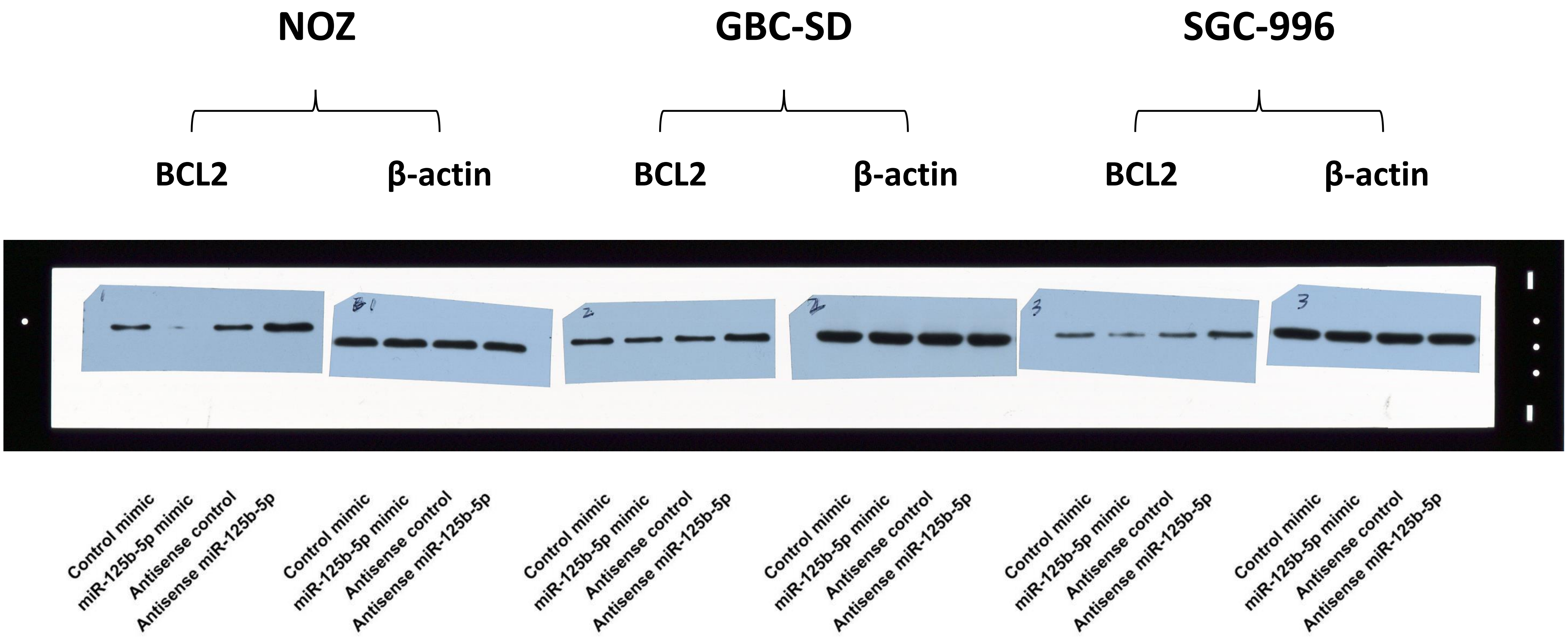

Supplementary Table 1 Primers of qPCR

Genes (*Homo sapiens*)	Primers	Sequences
	Anchor	CGACTCGATCCAGTCTCAGGGTCCGAGGTAT
	RT primer	TCGATCGAGTCGCACTTTTTTTTTTTTV
miR-125b-5p	Forward	5'-TCCCTGAGACCCTAACTTGTGA-3'
	Reverse	5'-AGTCTCAGGGTCCGAGGTATTC-3'
U6	Forward	5'-CTCGCTTCGGCAGCACA-3'
	Reverse	5'-AACGCTTCACGAATTTGCGT-3'
BCL2	Forward	5'-CAACATCGCCCTGTGGATGA-3'
	Reverse	5'-GGCCAAACTGAGCAGAGTCT-3'
GAPDH	Forward	5'-GAAGGTGAAGGTCGGAGTC-3'
	Reverse	5'-GAAGATGGTGATGGGATTTC-3'
